# Updates from the Intestinal Front Line: Autophagic Weapons Against Inflammation and Cancer

**DOI:** 10.3390/cells1030535

**Published:** 2012-08-21

**Authors:** Federica Madia, Valentina Grossi, Alessia Peserico, Cristiano Simone

**Affiliations:** 1 Laboratory of Signal-dependent Transcription, Department of Translational Pharmacology, Consorzio Mario Negri Sud, Santa Maria Imbaro (CH) 66030, Italy; Email: vgrossi@negrisud.it (V.G.); peserico@negrisud.it (A.P.); 2 Division of Medical Genetics, DIM, Faculty of Medicine, University of Bari, Policlinico, Piazza Giulio Cesare 11, Bari 70124, Italy

**Keywords:** Autophagy, colorectal cancer, inflammation, chemotherapy, p38α

## Abstract

The intestine lies at the interface between the organism and its environment and responds to infection/inflammation in a multi-leveled manner, potentially leading to chronic inflammatory pathologies and cancer formation. Indeed, the immune response at the intestinal epithelium has been found to be involved in the origin and development of colorectal cancer, which is the third most commonly diagnosed neoplastic disease. Among the mechanisms induced upon inflammation, autophagy appears as a defensive strategy for the clearance of invading microbes and intracellular waste components. Autophagy has also been found to play an important role in colorectal cancer, where it seems to have a pro-survival or pro-death function depending on the stage of the neoplastic process. In this paper we discuss the dual role of autophagy in colorectal cancer and review evidence showing that modulation of autophagy affects the immune response and cancer biology. The study of key players involved in autophagy might contribute to the design of new approaches for colorectal cancer, consisting in combined therapies capable of modifying cancer-specific metabolism rather than simply evoking a generic apoptotic and/or autophagic response, thus enhancing the efficacy of currently used drugs and treatments.

## Abbreviations

CRC: colorectal cancer
IBD: inflammatory bowel disease
UC: ulcerative colitis
CD: Crohn’s disease

## 1. Notes on Colorectal Cancer

### 1.1. Epidemiology and Genetics

It is estimated that in 2012 577,190 Americans (US) will die from cancer, corresponding to more than 1,500 deaths per day [1]. A similar scenario has been drawn for Europe [2]. Colorectal cancer (CRC) is the third most commonly diagnosed neoplastic disease, after those affecting the lungs and the prostate/breast, and the second leading cause of death among all cancer patients, with no apparent gender-related differences. Worldwide, about one million people each year develop CRC [3], which makes it one of the most deadly diseases in Western countries.

With a predominantly epithelial origin, CRC phenotypically consists of a broad spectrum of epithelial lesions ranging from adenoma to adenocarcinoma, the evolution of which ranging from benign growth to invasive stages often occurs over 10 to 20 years.

Though sporadic occurrences account for most CRCs (75%), epidemiological studies have highlighted a strong genetic contribution in the remaining 25% of cases, based on the observation that the incidence increases in patients with a personal and/or familial history of CRC. Only 5 to 6% of inherited cases are linked to specific genetic mutations. This is the case for major CRC genetic syndromes, which include familial adenomatous polyposis (FAP), attenuated FAP (AFAP), hereditary non-polyposis colorectal cancer (HNPCC) or Lynch Syndrome, MUTYH (mut-Y homolog) associated polyposis and rare hamartomatous polyposis conditions such as the Peutz-Jeghers (PJ) syndrome, the juvenile polyposis (JP) syndrome, the Cowden syndrome and the Bannayan-Riley-Ruvalcaba (BRR) syndrome. Indeed, the vast knowledge acquired over the past twenty years from studies regarding the above syndromes has provided significant clues over the genes and pathways leading to CRC.

Most sporadic cases of CRC (about 85%) are linked to chromosomal instability (CIN), allelic imbalance at different loci, chromosomal amplification and translocation, which mainly cause aneuploidy due to loss of portions of chromosomes 5q, 8p, 17p and 18q. For example, loss of function in the *APC* (adenomatous polyposis coli) gene on chromosome 5q, which encodes for a cell-adhesion protein and whose main downstream target is β-catenin, is the earliest event leading to transformation of colon epithelium and is also inherited in an autosomal dominant pattern in FAP and AFAP [4]. Loss of expression of *SMAD4* (SMAD family member 4), which is a critical component of the transforming growth factor β signaling pathway together with *SMAD*2 and 3, and is located on 18q, a frequent site of loss of heterozygosity (LOH), is linked to the transition from adenoma to high-grade carcinoma and is associated with the JP syndrome. Mutations in the *TP53* gene (on chromosome 17p), combined with LOH are also linked to transformation from adenoma to cancer and are linked to the Li-Fraumeni syndrome, inducing colon cancer among several others. Additionally, *KRAS* and *BRAF* mutations, which activate the MAPK cascade, are frequently associated with CRC (35%–45%) and are linked to the cardiofaciocutaneous syndrome.

The remaining sporadic cases, about 15%, are microsatellite instability (MSI) phenotypes, mostly due to mismatch (MMR) and base-excision (BER) DNA repair mechanism impairments, giving rise to frameshift or base-pair substitutions in short tandem-repeat sequences. This is the case for mutations in various genes (*MLH1, MSH2, MSH6*) often associated with CpG methylation-silenced promoter regions (*MLH1*) and in genes (*MINT1, MINT2, MINT3*) involved in NOTCH signaling. Interestingly, together with methylation most of the defects in MMR or BER genes specifically induce cancer initiation. The HNPCC syndrome is indeed linked to mutations in MMR genes, which are inherited in a dominant pattern and cause multiple primary CRCs, acceleration of tumor progression in case of somatic mutations, as well as other tumors [5]. Interestingly, germline base-excision repair defects in the *MUTYH* gene, which codes for a glycosylase involved in oxidative DNA damage repair, lead mainly to HNPCC or MYH-associated polyposis (MAP) with a very high incidence of CRC [6,7,8]. The MAP syndrome shows autosomal recessive inheritance and results from biallelic mutations in the MYH gene [9,10,11].

Besides CIN- or MSI-associated CRCs, several low-penetrant mutations have been observed, inducing rare syndromes. Germline mutations of the *LKB1/STK11* tumor suppressor gene on chromosome 19p, driven by LOH or by missense mutations and frameshifts, have been shown to cause the rare PJ syndrome [12]. Additionally, mutations in the tumor suppressor *PTEN*, which are frequently observed in tumors, are linked to the Cowden syndrome and to the BRR syndrome, a spectrum of conditions collectively known as *PTEN* hamartoma tumor syndromes.

### 1.2. Environmental Risk Factors in CRC Development

It is becoming increasingly clear that initiation and progression of CRC from normal epithelium to early adenoma (premalignant), to invasive adenocarcinoma and then to metastatic disease is determined by a multi-step sequence of molecular events—some of which have been briefly described above—including germline mutations and often acquired mutations, which usually occur at specific stages. For instance, mutations in the *APC* gene occur early and are often followed by mutations in *RAS*-family genes, whereas *TP53* changes tend to occur at later stages [13,14,15]. This gives rise to dynamic processes, the genetic contributions of which interconnect with each other and with the environment.

Aging, for instance, has been shown to be a predominant risk factor with more than 90% of CRC cases occurring in people aged 50 or older. This relationship is exacerbated in the progeroid Bloom’s syndrome, which leads to premature aging. In this syndrome, mutations in the BLM gene, which encodes for a DNA helicase repair enzyme, are associated with multiple malignancies, including CRC [16,17]. Cancer development and disease prognosis are also heavily affected by environmental factors, with unhealthy lifestyles, including lack of regular physical activity, low-fiber and high-fat diet, overweight and obesity (that is, all sources and/or symptoms of metabolic imbalance), alcohol consumption and tobacco use [18,19,20,21,22], being the most relevant.

Additionally, a crucial role is played by the intestinal environment, which is involved in signals and events leading to CRC. A complex microbial system colonizes the healthy human intestine, affecting host physiology and host health. Intestinal microflora has been shown to directly and indirectly contribute to the development and recurrence of inflammatory diseases by modifying the chemical status of the environment (*i.e.*, pH, O_2_ levels and release of toxins), exacerbating the immune response, affecting the intestinal epithelium both functionally and structurally and predisposing the intestinal tract to CRC development and progression [23,24,25].

## 2. Role of Autophagy in Intestinal Inflammation and Colorectal Cancer

### 2.1. From Inflammatory Disorders to Neoplasia

The immune response at the intestinal epithelium, which lies at the interface between the organism and its environment, has been shown to play a pivotal role in the origin and development of CRC, reinforcing the hypothesis that this type of cancer is the result of complex interactions [5,26,27].

CRC predisposition has been observed in patients with ulcerative colitis and Crohn’s disease, which are typical forms of inflammatory bowel disease (IBD). Chronic mucosal inflammation positively correlates with increased occurrence of malignancies, also in association with preceding inflammatory disorders [28], and predisposes to cancer with age-related increasing incidence in almost 25% of patients. It must be noted that not only classical IBD, but also other types of colitis have a high chance of developing to CRC [29]. Cytokines released by epithelial and immune cells have a role in the pathogenesis of colitis-associated cancer. The role of cytokines can be either pro-inflammatory, tumor-promoting (interleukins IL-6, IL-12 family, IL-23) or tumor-inhibiting, as is the case of IL-10 [30]. For example, tumor necrosis factor-alpha (TNF-α), which is released by immune cells, has been shown to promote cancer in experimental colitis. By binding to its receptor TNF-R, TNF-α affects the activity of NF-kB, one of its many downstream effectors, and can promote DNA damage, stimulate angiogenesis and induce expression of cyclooxygenase-2 (COX-2) [31].

In this scenario, oxidative stress in the form of oxygen and nitrogen radicals (RONS) induced by inflammatory cells can affect the activity of signaling pathways (COX-2, prostaglandin E_2_), the expression of genes encoding for transcription factors (NF-kB) as well as of tumor suppressors (p53) and MMR and BER DNA repair genes [26,28,32]. In fact, patients bearing defects in ROS pathways have a predisposition to colitis [33]. It is worth noting that modifications in arachidonic acid metabolism leading to the activation of prostaglandin signaling have been shown to represent a critical step in the development of adenomas. Moreover, COX-2, which mediates the synthesis of prostaglandin E_2_, is constitutively over-expressed in skin and colorectal tumors [26,34].

Inflammation and modifications of intestinal cells are linked to the activation of epidermal growth factor (EGF) signaling. EGF activates downstream pathways such as the MAPK and PI3K cascades, thus reinforcing tumor growth and progression [5]. Besides, the PI3K/Wnt pathway is associated with COX-2/PGE₂ production [35]. Also, the activity of endothelial cells is strictly correlated to RONS- and oxidative stress-dependent induction of inflammation. Resulting cellular adjustments and modifications modulate vascular supply and immune cell migration and mediate the release of growth factors, such as the vascular endothelial growth factor (VEGF), cytokines and chemokines; moreover, they are also involved in the up-regulation of NF-kB, thereby affecting the propagation and evolution of diseases such as IBD [36]. Biopsy samples of IBD patients have revealed activation of NF-kB specifically in enterocytes and macrophages from the lamina propria [37]. Increased inflammation in a 1,2-dimethylhydrazine-induced CRC model has also been reported to trigger the induction of the transcription factor NF-kB. Its activation promotes growth signaling through the regulation of both pro-inflammatory proteins [inducible nitric oxide synthase (iNOS) and COX-2] and pro-inflammatory cytokines (TNF-α and IL-6), and through the over-expression of zinc family metalloproteinases (MMP, 2–9) [38,39]. The production of cytokines such as IL-6 also initiates, through activation of NF-kB, a signaling pathway comprising signal transducers (STAT3) which in turn promote growth of colitis-associated cancer.

Interestingly, activation of both NF-kB and STAT3 often leads to the expression of anti-apoptotic, pro-proliferative and immune response genes in the tumor cell microenvironment as well as in immune cells infiltrating it, but opposite responses can be observed from different cell types. Anti-apoptotic signals and/or inactivation of tumor suppressors are usually associated with epithelium transformation, but depending on the inflammation state, or on the specific pathway activated, either anti-apoptotic or pro-apoptotic signals can be triggered [40,41]. Thus, the intestinal epithelium may respond to the initial infection/inflammation in a multi-level manner, leading to chronic inflammatory pathologies and cancer formation. Massive changes occur in the intestinal environment with the activation of overlapping signaling pathways associated with increased oxidative stress and increased epithelial, endothelial and immune cell infiltration. This in turn induces the release of cytokines and chemokines, thus boosting a stronger immune response and giving rise to a vicious circle.

### 2.2. Autophagy as a Line of Defense to Environmental Insults: The Immune System Strategy

The first immune response evoked against toxins, pathogens or modifications in the population of residential intestinal microbes can rely on autophagy as a defensive strategy for the clearance of invading microbes as well as intracellular waste components such as organelles and apoptotic bodies, and for supporting survival in a mutable environment [42,43].

Autophagy is a highly conserved cell degradation pathway involving the sequestration of cytoplasmic components within double-membrane vesicles named autophagosomes, which are then delivered to lysosomes. Its activation is essential to generate intracellular nutrients to help cells efficiently maintain high metabolic levels, and provides energy for proliferation in states of high bioenergetic demands, as is the case during infection/inflammation. This process also occurs during tissue remodeling and under cellular stress conditions such as nutrient deprivation, upon which protein recycling provides the energy required for survival [44].

Autophagy is not unique to stress conditions; virtually all normal and healthy cells use this process to maintain a balance between synthesis, degradation and subsequent recycling of cellular structures [45]. At the biomolecular level, it has been described as a multi-step series of events—initiation, elongation, cargo selection and autophagosome maturation—all mediated by the activation of kinase cascades and ubiquitin-like machinery.

The first autophagic step is triggered by the action of key proteins involved in the recognition of pathogens: NOD2, which binds to the bacterial cell wall; IRGM, which plays multiple roles in pathogen elimination, autophagy induction and maturation; VDR (vitamin D receptor) and DAP, which directly respond to nutrient availability, energy supply, bacteria *etc*. [46,47]. Orchestrated activation of the ULK (uncoordinated-51-like kinases) kinase complex and recruitment of the class III PI3K complex (Beclin-1, Vps34, serine/threonine kinase p150 and Barkor/mATg14) initiate the process. Elongation occurs through ubiquitin-like conjugation systems involving Atg7, Atg10 with Atg12, Atg5 and Atg16L1 together with Atg4, Atg7, Atg3, and modification of the LC3 (microtubule-associated protein light chain 3) complex, after which cargo recruitment is promoted. In this phase, the adaptor proteins p62/sequestosome 1 (SQSTM1) and NBR1 and NDP52 recognize target ubiquitinated substrates and bind them to the LC3 complex. Finally, maturation of autophagosomes and fusion to lysosomes occur, mediated by the small GTPase Rab7 and by LAMP-1 and -2 [48,49].

Over the past decade, this mechanism has been identified as playing a primary role in the prevention and causation of diseases. Indeed, studies have shown that imbalance of the autophagic machinery is associated with a variety of human pathologies including infections, neurodegeneration, aging, heart diseases and cancer.

In the complex etiology of IBD, for example, in which various factors combine to cause chronic inflammation in the gastrointestinal tract, it is becoming increasingly clear that many of the genetic factors involved are implicated in autophagy.

Patients affected by Crohn’s disease (CD) show impaired autophagy. Specifically, CD susceptibility has been associated with autophagic genes (*ATG16L1, IRGM, NOD2* and *LRRK2*). A two-fold increase in disease risk has been reported in patients homozygous for a single nonsynonymous SNP (single nucleotide polymorphism) in the coding region of *ATG16L1*. Specifically, an amino acid change from threonine to alanine at position 300 (T300A) within the WD-repeat region has been reported to induce alterations of protein-binding partners crucial for autophagy, rendering the region dispensable for starvation-induced autophagy. Defects associated with the T300A region variant of *ATG16L1* are also proposed to impair antibacterial autophagy [50,51]. Indeed, a crucial role for *ATG16L1* in antibacterial autophagy has been confirmed in several *in vitro* and *in vivo* models, which showed a cell type-dependent impairment of autophagy in various types of cells, including lymphoblasts, macrophages and dendritic cells from CD patients. *In vivo* studies in *ATG16L1* KO mice (obtained by fetal liver chimeric ablation of the gene, which induces its functional knockout in the hematopoietic system) used in a DSS-induced colitis model demonstrated a specific role for this protein and its related autophagic pathway in the regulation of pro-inflammatory cytokine production in response to bacterial stimulation, with hypersensitivity of the immune system. In models where *ATG16L1* expression was reduced to 30% of normal values, features of CD epithelium alterations were evident, such as morphological changes in secretory Paneth cells. These cells showed adiponectin deposits, although no increase in inflammation was observed [52].

Mutations in the *NOD2* (nucleotide-binding, oligomerization domain 2) gene, which encodes for a bacterial protein sensor of the NLR (NOD-like receptor) family, are associated with CD susceptibility. Three variants are most commonly involved—two missense mutations, 702 (arginine to tryptophan) and 908 (glycine to arginine), and one frameshift caused by a cytosine insertion at position 1007—which all induce a high reduction or even loss of function of *NOD2*. The risk of CD increases 2.4-fold and 17.1-fold in individuals heterozygous and homozygous, respectively, for these variants. NOD2 is involved in the first response to pathogens, being the ligand of the bacterial cell wall peptidoglycan. Its activation triggers MAP kinase cascades, which in turn affect transcription of NF-kB, hence inducing cytokine expression. NOD2 activation also induces NO production, thereby increasing immune response. *NOD2* showed tight interactions with the autophagic *ATG16L1* gene, thus reinforcing the idea that IBD susceptibility arises from dysregulation of the autophagic pathway rather than of a single gene [53,54]. Studies in primary immature dendritic cells and in the intestinal epithelium have clearly shown that NOD2 activity requires the activation of different Atg proteins. Interestingly, studying the genotypic basis of ulcerative colitis and Crohn's disease by overlapping clinical and epidemiological features, Waterman and colleagues found divergences in NOD2/autophagy pathway SNPs, but similarities in most IL-22/23 Th17, adaptive immunity, and barrier pathways SNPs [55].

**Figure 1:**
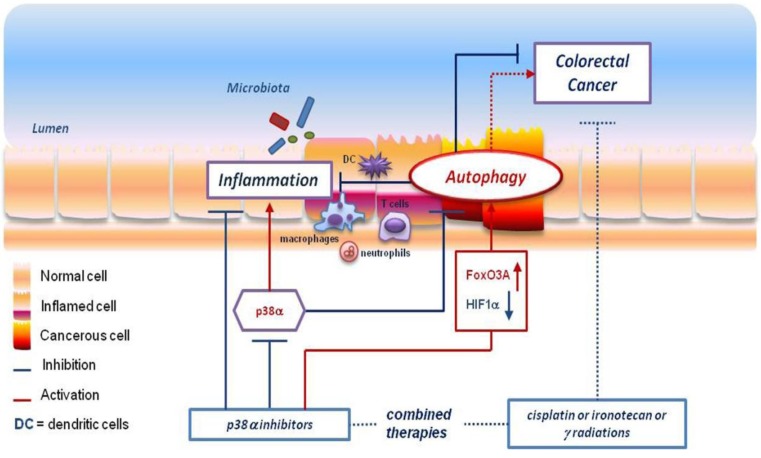
p38α inhibition: A new approach to fight inflammation and colorectal cancer. Autophagy plays a crucial role in the intestine, where it can have a pro-survival or a pro-death effect, acting as a defense/attack strategy in inflammation/immune related diseases and in colorectal cancer development. In this context, manipulation of p38α, one of its key players, may represent a promising tool to fight both inflammatory diseases (*i.e.*, IBD) and cancer. The p38α inhibition attenuates cytokine secretion, thus reducing inflammation, and in combined therapies it has been shown to enhance the efficacy of currently used drugs. Indeed, inhibition of p38α promotes FoxO3A-dependent gene expression, inhibits HIF1α activity and boosts the autophagic response.

Further genetic defects in various genes, with primary or secondary roles linked to host defense, were found to be general risk factors for CD and IBD. Several studies described the effect of IRGM mutation variants, LRRK2 or ULK1 complex mutations or VDR polymorphisms in immune response, autophagy and susceptibility to CD or IBD. IRGM, for example, is essential for the elimination of various pathogens. Genome-wide association studies have identified multiple CD-associated SNPs in the IRGM locus [56,57]. *ULK1*, which is regulated by the AMPK and m-TOR pathways, is essential for autophagy initiation, and its impairment can have a broad effect on the autophagic process in all tissue districts. *LRRK2* defects have been linked to autophagy impairment and associated not only to CD but also to leprosy and Parkinson’s disease [54]. VDR polymorphisms have also been linked to IBD susceptibility [58,59,60]. A direct association with altered autophagy induction has not been established for this factor, but vitamin D plays a role in various steps of the autophagic process and could thus be indirectly involved in IBD pathologies. On the other hand, the expression of *NOD2* has been recently shown to be regulated by *VDR* [61]. The studies outlined above suggest a tight link between defects in the autophagy defensive strategy and susceptibility to IBD. Furthermore, as previously mentioned, IBD and chronic inflammation are associated with colorectal cancer, at least in the case of colitis-associated CRC. Therefore, it is not surprising that in CRC tissues, normal epithelial, malignant, endothelial and immune cells, macrophages and the microflora, that all lie in the same environment, share similar pro-survival, pro-growth or pro-death signaling, with autophagic pathways being among them (Figure 1).

### 2.3. Autophagy as a Tactic to Survive: The Colorectal Cancer Strategy

In cancer cells, autophagy is activated to counteract rapid environmental changes, in response to high energetic demands, as a defensive strategy against immune response, in the initial transformation or mostly as a late adaptive response. However, the question of whether autophagy leads to cancer cell killing or to cancer cell protection against disadvantageous conditions is yet to be answered; this is partly due to the observation that autophagy shows a heterogeneous spatial- and function-dependent distribution in the normal intestinal epithelium, and its contribution varies depending on the stage of the neoplastic process.

In normal colon mucosa, significant levels of autophagic activity, which supposedly exert a crucial cytoprotective role, have been detected in the undifferentiated proliferative compartment of the crypt and in the progenitor/stem cell population of the colonic gland when compared to the upper portion of the superficial epithelium consisting of more differentiated/quiescent cells [62].

At early stages of tumor development, autophagy has been reported to act as a tumor suppressor process. Supporting evidence has been obtained in different cell lines from breast, ovarian and colon cancer in which this mechanism is inhibited, resulting in developmental and growth advantages. These consisted not only in reduced protein degradation, but also in increased mutations caused by oncogenes or stress conditions, which would otherwise be eliminated by the autophagic machinery [10]. Reduced levels of autophagy correlate with tumorigenesis, and several inducers of autophagy have been found to be tumor suppressors. Several tumor suppressor proteins, including the BH3-only proteins; DAPK1 (death-associated protein kinase-1); PTEN, a phosphatase that antagonizes PI3K; TSC1 and TSC2 (tuberous sclerosis complex 1 and 2); and LKB1/STK11 induce autophagy, while their loss is associated with reduced autophagy and correlates with cancer development [48,63]. As in IBD, one of the first genes found to be linked to autophagy in CRC is the autophagic inducer Beclin1. In fact, decreased expression levels of the *BECN1* gene have been detected in many breast cancer specimens. Interestingly, Knævelsrud and colleagues have observed that one third of MSI-associated CRCs are linked to mutations in the *UVRAG* gene, which codes for a Beclin 1-binding protein that is a positive regulator of the class III PI3K/Vps34 complex. UVRAG has been implicated in the formation and maturation of autophagosomes as well as in endocytic trafficking, suppression of proliferation and *in vivo* tumorigenicity [64]. However, inhibition of *UVRAG* or its over-expression in wild-type cells does not affect the autophagic process, thus suggesting that the involvement of this gene in the MSI phenotype relies on mechanisms other than autophagy [65]. Interestingly, recent evidence has suggested that autophagy is required during malignant transformation. It has been demonstrated that pharmacological inhibition of autophagy completely blocks Ras-mediated anchorage-independent cell growth on soft agar, therefore attenuating the transformed phenotype [66]. Similarly, genetic ablation of autophagic genes (*ATG* genes) suppresses the transforming ability of Ras, thus suggesting that activated oncogenes require autophagy to promote tumorigenesis [67].

Along tumor progression, autophagy acquires a leading role as a tumor cell protective mechanism against newly arising conditions. In this stage, dramatic changes occur in cellular function, signaling and environment and cells display differential energy demands also depending on their spatial position in tumor architecture. Peripheral cells can benefit from higher nutrient and oxygen availability, as they are closer to the bloodstream, whereas inner cells face more stringent and stressful starving-like conditions; yet, both cell types might rely on autophagy. Particularly high induction of autophagy has been observed in CRC during cancer progression as well as in nutrient-deprived conditions [68]. Indeed, inhibition of mTor through rapamycin treatment or blockage of the PI3K-AKT-mTOR oncogenic pathway, which is activated in several malignancies including CRC, mimics starvation conditions and promotes autophagy; similar evidence has been obtained also in yeast, *C. elegans* and *D. melanogaster* [69,70].

Interestingly, in glucose-deprived conditions CRC cell survival is also supported by adiponectin. This protein exerts a key role in the positive correlation between obesity and cancer through the enhancement of autophagy mediated by activation of AMPKα and PPARα and inhibition of the IGF-1/PI3k/Akt/mTOR pathway [71].

Other common oncogenes have been described to regulate autophagy, including anti-apoptotic proteins of the Bcl-2 family, death-associated/related protein kinases (DAPK; DRP1), HSP family proteins and mitogen-activated kinases (MAPKs). In particular, evidence obtained in HT-29 CRC cells revealed induction of autophagy through the extracellular signal-regulated kinases ERK1 and ERK2, when stimulated by the RAS-RAF-MEK pathway [72]. It is thus clear that a complex network of molecular events is involved in the autophagic response and this mechanism acquires different roles throughout the CRC development.

### 2.4. Autophagy and Apoptosis: Dual Role in Colorectal Cancer

In CRC cells autophagy indeed acquires a crucial antagonistic role to apoptosis. While the autophagic response can be elicited as a protective mechanism to boost energy demands and degrade damaged organelles, or as a defense against the immune response, at later stages, when intracellular damages reach a detrimental threshold, autophagy may contribute to programmed cell-death (PCD), with features similar, but not identical, to those of apoptosis [10,73]. Depending on cancer stage and conditions, these two mechanisms are promoted interdependently. In experiments where nutrient starvation (amino acids or glucose) is applied to several CRC cell lines, autophagy is highly induced, while apoptosis is enhanced after treatment with autophagy inhibitors such as the type III PI3K inhibitor 3-methyladenine (3-MA) or upon lowering of *ATG7* expression [74].

Indeed, CRC cells may arise through the accumulation of genetic defects that affect apoptosis regulation. Mutations in the *APC* tumor suppressor gene are the initiating lesion, and the consequent activation of the Wnt signaling has been shown to drive cancer progression. This latter pathway induces early massive apoptotic events in the epithelium, but cells escaping apoptosis survive through autophagy and may lead to the formation of populations capable of evolving into adenoma by counteracting the defensive signaling of the host immune system [75]. Interestingly, evidence has been reported that autophagy negatively correlates with the activity of Wnt signaling in CRC progression [76].

A fine balance between autophagy and apoptosis seems to be the winning strategy for cell survival in changing conditions. In this context, as previously mentioned, tumor cell architecture and nutrient and oxygen availability may have interrelated effects capable of triggering either response. The antagonism between apoptosis and autophagy is well represented by the action of the NOD1/CARD4 and NOD2/CARD15 members of the Nod-like receptor family. These proteins are located in the cytosol; they bind bacterial and viral ligands and play a key role in innate and adaptive immune response, apoptosis, autophagy and ROS generation. Polymorphisms in NOD1/CARD4 and NOD2/CARD15 genes may shift the balance between pro- and anti-inflammatory cytokines, thus modulating the risk of infection, chronic inflammation and cancer (e.g., CRC) [77].

Interestingly, recent data obtained by Livesey and coworkers suggest that the tumor suppressor p53 is at the crossroad between this dual role. The authors reported that p53 forms a complex with the high mobility group box1 (HMGB1) protein, a damage-associated molecular pattern molecule (DAMP) that regulates cell death and cell survival and increases autophagy by sustaining Beclin-1-PtdIns3KC3 complex activation and by interacting with RAGE (receptor for advanced glycation end products) [78,79]. The p53-HMGB1 complex then regulates the cytoplasmic localization of the Beclin-1-PtdIns3KC3 complex-binding protein and consequently modulates autophagy levels [79]. p53^−/−^ HCT116 human CRC cells display increased HMGB1 expression and induction of autophagy, whereas HMGB1^−/−^ mouse embryonic fibroblasts show p53 over-expression and apoptosis induction. In a previous work, however, loss of p53 in the HCT116 CRC cell line has been reported to impair autophagy upon starvation and to induce cells to undergo apoptosis [80]. Interestingly, p53 has been shown to inhibit autophagy in colon adenocarcinoma LoVo cells by interacting with RB1CC1/FIP200 (ortholog of yeast Atg17), a protein involved in the initial steps of autophagy [81].

Chronic inflammation-induced oxidative stress might also contribute to tumor transformation. Indeed, RONS interact directly with the DNA in the proliferating epithelium resulting in genomic alterations and thus contributing to p53 mutations, MMR DNA repair mechanism impairment and even mitochondrial damage; hence, oxygen levels and glucose might also contribute to regulating autophagy and/or apoptosis. In this regard, CRC cells have been shown to trigger autophagy not only for the degradation of damaged mitochondria to prevent release of pro-apoptotic factors, such as cytochrome c (cytC) [82], but also in response to intrinsically highly activated aerobic glycolysis [83].

### 2.5 Regulators in the Autophagic Response of Colorectal Cancer

Cancer cells may indeed benefit from their ability to utilize aerobic glycolitic pathways for energy generation while down-regulating mitochondrial respiration. This phenomenon, described by Otto Warburg [84], is evolutionarily conserved from lower eukaryotes to humans, allowing cells to adapt to different situations [85] and is based on the specific up-regulation of catabolic reactions such as glycolysis and glutaminolysis. The glycolitic process generates energy much less efficiently than mitochondrial respiration (two ATPs/glucose compared with 38 ATPs/glucose), thus glucose uptake is increased while glucose consumption is lowered. Intriguingly, glycolysis induction normally occurs in hypoxic conditions when O_2_ concentration decreases to roughly 1% (normal levels are *ca*. 21%), but it can also take place in normoxia conditions. Cancer cells rely on this phenomenon to either proliferate in a hypoxic environment or to escape apoptosis by down-regulating the oxidative metabolism despite oxygen availability [86]. It is worth noting that some hepatoma, breast and glioma cancer cells showed highly functional activated mitochondria [87,88,89].

In CRC, deregulation of cell metabolism can stimulate autophagy. There is evidence indicating that glycolytic enzymes such as GAPDH positively regulate autophagic genes and therefore indirectly affect autophagy. Conversely, glutaminolysis regulates autophagy in a direct manner by producing ammonia, which is known to increase the autophagic process [90]. A very high glucose uptake has been observed in CRC, and the p53 status has been suggested to be one of the factors partially responsible for the Warburg effect, promoting or inhibiting oxygen utilization [83,91]. In this scenario, the hypoxia-inducible factor HIF-1 (HIF-1α-HIF-1β complex) acts as a master regulator of the Warburg effect and is responsible for the induction of more than one hundred genes that facilitate adaptation and survival, including glucose transporters, glycolytic, rate-limiting and pyruvate-lactate conversion enzymes as well as the tumor suppressors p53, p21, the apoptotic factors caspase-3 and caspase-9, the cell proliferation factors IgF2, TGF-α, the angiogenesis factors NOS2, HO-1, endothelin-1 and immune factors [92].

It is then not surprising that regulation of HIF-1α and its target genes is essential in CRC cells, which display high energy demands and are controlled by an intricate network of signaling pathways (PI3K/PTEN/Akt, JNK cascade, Wnt, p53, K-ras, LB1/AMPK, TGF-β). HIF-1α represents a strong link between aerobic glycolysis and carcinogenesis by virtue of its ability to sense nutrients and oxygen for survival [93]. HIF-1α activity regulators or interplaying factors, such as the MAPK pathway, are indeed up-regulated in colonic tumorigenesis. Several other genes, including those encoding for p21-rac1, MAPK p38α, rho-GDP dissociation inhibitor 2, p21-activated kinase α and MAPK 6, have been shown to be up-regulated even in low-grade dysplastic adenomas [93]. The p38α MAP kinase, one of the four isoforms (α, β, γ and δ) of the p38 family, belongs to a class of serine/threonine MAPKs [94,95] and is required, together with isoform β, for breast and colorectal cancer cell migration and metastasis formation in mice. It has been shown to sustain the expression of HIF-1α target genes in CRC and ovarian cancer cells (OvCa), providing a considerable and necessary boost to cellular proliferation and survival in a cell type-specific manner, regardless of the malignant phenotype and genotype [96,97,98,99]. Furthermore, p38α MAP kinase inhibition causes a drastic decrease in ATP intracellular levels in CRC and OvCa cells. Prolonged inactivation of p38α by pharmacological blockade of its kinase activity with the specific inhibitor SB202190 triggers the AMPK-mediated activation of FoxO-dependent transcription, leading to autophagy, cell cycle arrest and cell death in both cancer cell types. FoxO3A ablation by specific RNAi significantly inhibits SB202190-dependent autophagic response, thus confirming the role of this transcription factor in this process [99]. *In vivo*, pharmacological blockade of p38α inhibits CRC growth in xenografted nude mice and azoxymetane (AOM)-treated APC^Min/+^ mice, achieving both a cytostatic and cytotoxic effect. These studies revealed that p38α blockade inhibits CRC cancerogenesis by inducing a transcriptional switch from HIF1α- to FoxO3A-dependent gene expression, resulting in a shift from glycolysis genes to genes involved in fatty acid catabolism and protection against stress conditions [97,100,101].

These findings further support the tight relationship between inflammatory diseases and CRC, also in light of the role played by the HIF-1α transcription factor, the activity of which has been linked to the immune response. Specifically, HIF-1α deficiency has been shown to induce dramatic defects in B lymphocyte development and autoimmunity, and IL-1 and TNF-α have been found to highly trigger HIF-1α expression [102,103,104]. Moreover, enhanced HIF-1α levels, DNA-binding and gene expression have been observed in macrophage cultures upon bacterial lipopolysaccharide increase [102]. The transcriptional shift from HIF-1α- to FoxO3A-dependent gene expression promoted by p38α blockade indirectly shows that HIF-1α suppression could prove beneficial to prevent tumor growth, angiogenesis and cell adaptation to hypoxia or to energy depletion in CRC, even though its activation would be desirable in the first phases of the immune response.

Autophagy is induced both in normal and malignant cells in response to diverse stimuli such as starvation, growth factor deprivation, hypoxia and toxins (including therapeutic agents) in a cell type-specific and signal-dependent manner. The resulting outcomes vary from protection and survival to cell growth arrest and programmed cell death. This mechanism essentially represents a survival response to metabolic stress that, under persistent stress conditions, can also result in cell death.

Recent observations indicating that some types of cancer cells, including CRC, display features of non-apoptotic cell death (*i.e.*, autophagic cell death or PCD II), in response to chemotherapy or γ-irradiation, are in contrast with others indicating that solid tumors defective in apoptosis rely on autophagy to survive under metabolic stress conditions. Despite its controversial role, autophagy manipulation is becoming a promising tool to fight cancer; however, associated risks must be carefully considered [10,46].

The overall scenario highlights the extreme difficulties still being faced by the research community in designing a successful strategy for cancer treatment. This is especially true for CRC, the etiology of which is linked in a multi-level fashion to chronic inflammation and to inflammatory bowel disease (in its two forms, ulcerative colitis and Crohn’s disease), disorders where autophagy has a crucial defensive role.

## 3. Modulation of Autophagy: New Strategies to Fight CRC

### 3.1. Current Therapies

Treatments based on combined therapies, including surgery, local radiotherapy and chemotherapy are currently available for CRC. Treatment type and timing vary according to tumor stage classification, and the success of surgical resection often depends on effective pre-operative tumor staging, which is aimed at accurately defining tumor penetration, lymph-node involvement and the existence of distant metastatic disease. A variety of pre-operative therapies can be used before surgery on the basis of pre-operative staging. Potential advantages of a pre-operative approach include decreased tumor seeding during surgery, reduced toxic effects, increased radiosensitivity due to more oxygenated cells and a potential for sphincter preservation.

The chemotherapy options available for metastatic CRC patients are combinations of 5-fluorouracil/leucovorin (5-FU/LV) and irinotecan (FOLFIRI) or 5-FU/LV and oxaliplatin (FOLFOX) associated or not with the anti-VEGF antibody Bevacizumab or the anti-EGFR antibody Cetuximab. Cetuximab indication is restricted to patients with wild type KRAS, since KRAS mutations predict resistance to treatment with anti-EGRF monoclonal antibodies.

Nowadays, pre-operative chemo- plus radiotherapy followed by surgery and adjuvant chemotherapy provides the best local disease control, sphincter preservation and surgical outcomes in locally advanced rectal cancer; however, CRC has a high percentage of metastatic disease and still shows a five-year survival rate of less than 60% [3].

### 3.2. New Approaches

The efficacy of current cancer therapies has been essentially relying on their DNA-damaging ability, which is expected to promote apoptosis. However, DNA mutations or functional imbalance of relevant DNA damage-dependent pathways involving apoptotic and/or anti-apoptotic genes cause the acquisition of drug resistance in several types of tumors, including CRC [98]. The potential of autophagy manipulation to synergize with other chemotherapies for the effective treatment of CRC is corroborated by a plethora of recently published studies.

Pharmacological inhibitors such as 3-MA, bafilomycin A1 (BafA) and chloroquine (CQ) have been widely used to inhibit autophagy. 3-MA is an inhibitor of class III PI3K Vps34, acting in autophagosome isolation membrane formation. Hence, 3-MA inhibits autophagy at an early stage, the sequestration step. In contrast, BafA, a vacuolar H^+^-ATPase inhibitor, prevents lysosomal function, thus blocking autophagosome degradation, a late stage of the process. Similarly, CQ, a weak base that can be trapped in acidic vesicles and elevates intralysosomal pH, also blocks cargo degradation [105,106,107,108,109].

Promising data come from different studies investigating autophagy inhibitors to enhance the anticancer activity of 5-FU, the best standard treatment for CRC. 5-FU exerts its therapeutic effect by inducing apoptosis. However, autophagy activation has been observed upon this therapy. In this context, several pieces of evidence underline the potential of combined treatment. Inhibition of autophagy with 3-MA has been shown to enhance 5-FU-induced CRC cell apoptosis and to improve the chemotherapeutic effect of 5-FU in the HT29 and HT26 CRC cell lines. Similar results have been obtained with the combination of 5-FU and chloroquine [110,111].

On the other hand, a recent study demonstrated that CRC cells defective in apoptosis use autophagy as an alternative cell death pathway [112]. These data suggest a paradoxical role of autophagy as a target for adjuvant therapy. The induction of autophagy has also been observed in CRC cells following treatment with cancer therapeutics, including histone deacetylase inhibitors (SAHA, vorinostat) and ionizing radiation [113].

A recent study showed that inhibition of autophagy with CQ strongly enhances vorinostat-mediated apoptosis in CRC cells. Indeed, the combination of CQ with vorinostat improves superoxide generation, ubiquitinated protein accumulation and apoptosis induction *in vitro* and *in vivo* in a CRC xenograft model [114].

Moreover, accumulating evidence supports the use of gene therapy to modulate autophagy. This approach might be desirable in combination with radiation therapy. CRC cells counteract radiotherapy through the induction of transcription of autophagy-related genes (Beclin-1, Atg3,4b/c,5,12). It has been shown that the combination of radiotherapy with short-interfering RNAs targeted against these genes enhances radiotherapy cytotoxicity in resistant cancer cells [115,116]. Although at present gene therapy is considered impractical in clinical settings, in the future it might be useful to improve cancer therapy.

The efficacy of various natural products has also been tested. Flavonoids such as quercetin, dietary phytochemicals such as curcumin or genistein in combination with indol-3-carbinol, silibinin, a flavonolignan isolated from the milk thistle plant (*Silybum marianum*), the cell wall skeleton of *Mycobacterium Bovis* Bacillus Calmette-Guerin (BCG/CWS) and several others have been recently reported to be effective in inhibiting or enhancing the autophagic response in CRC cells [117,118,119,120,121].

Based on the encouraging results from the above and from more recent studies, approaches designed to affect CRC pathophysiology by modifying its specific metabolism, rather than simply evoking a generic apoptotic and/or autophagic response, are highly desirable. In this perspective, manipulation of the p38α MAP kinase represents a promising tool for cancer therapy.

p38α pharmacological blockade not only induces autophagy in CRC *in vitro* and *in vivo* models, but also negatively affects HIF1α activity and promotes FoxO3A-dependent gene expression [99,100,101,122]. Importantly, it has been recently shown that FoxO3A mediates the cytotoxic effects of cisplatin, one of the therapeutic agents used in colorectal and ovarian cancer therapy [123]. In agreement with these data, p38α inhibition has been shown to positively reduce tumor growth when combined with cisplatin (Matrone A, Germani A, Grossi V, and Simone C, unpublished data). Interestingly, Paillas and coworkers have recently observed enhanced activation of the p38α MAP kinase in an *in vitro* drug-resistant CRC model (irinotecan drug metabolite SN38-resistant clones). The authors also detected increased cytotoxic activity of the drug metabolite SN38 upon p38α/β pharmacological blockade by SB202190 [124]. In another elegant co-culture model of *in vivo* “tumor setting”, where HCT116 CRC cells were cultured with supernatants from differentiated or activated macrophages, Bajbouj and colleagues observed enhanced p38 phosphorylation upon death-associated protein kinase (DAPK)-dependent apoptosis induced by TNF-α. As DAPK is involved in pro-apoptotic signaling upon cytokine exposure—hence during inflammation—and p38α has been identified as a novel DAPK-interacting protein, these data confirm the role of p38α in tumor cell death caused by immune cells [125]. Interestingly, p38α inhibitors are also in clinical trials for the treatment of inflammatory diseases, based on their potential to attenuate cytokine secretion [126] (Figure 1).

Autophagy up-regulation could be a useful tool not only for inhibiting CRC at early stages of development, where it acts as a tumor suppressor mechanism [10], but also for increasing the efficacy of the immune response in case of infection or inflammation caused by IBD. Encouraging evidence comes from studies on *H. pylori* infection treatments based on a combination of anti-oxidant, anti-bacterial and anti-adhesion agents. This approach was found to favor autophagy over apoptosis and proved effective to reduce inflammation, thus being also potentially beneficial for the prevention of gastric cancer [127]. Also, daily administration of aspirin has been proposed to decrease CRC incidence in patients with Lynch Syndrome [128] or in case of polyposis and intestinal inflammation, but the outcomes of this therapy are still under debate. Heterogeneous results have been obtained across studies and dose-risk and duration-risk relationships are still unclear [129,130,131]. In an effort to elucidate the mechanisms involved, Din and coworkers showed that the protective action of aspirin against the development of CRC is due to inhibition of the mTor-signaling effectors S6K1 and 4E-BP1, changes in nucleotide ratios and activation of AMPK, which result in increased autophagy [132].

## 4. Conclusion

The finding that autophagy significantly contributes to the economy of the intestinal epithelium, appearing as a common survival strategy for several cell types within the colon mucosa, has furthered the understanding of chronic inflammation, IBD and CRC development, shedding light on how these conditions are related to each other. In a complex structure such as the intestine, which is at the front line between the organism and its environment, the autophagic machinery plays a central function and its imbalance can trigger multi-level responses. Autophagy shows a heterogeneous spatial- and function-dependent distribution in the normal intestinal epithelium and its effect during inflammation or CRC growth appears to be beneficial or detrimental depending on the stage of the disease. Indeed, at early stages of cancer development it acts as a tumor suppressor process, while at later stages it acquires a primary role as a protective mechanism for tumor cells. In this context, new therapeutic tools for both inflammation and cancer might arise from in-depth investigations focusing on autophagy manipulation, with particular attention to the timing at which it will occur. Similarly, the study of key players involved in autophagy might also contribute to the design of new approaches for CRC, consisting in combined therapies capable of enhancing the efficacy of currently used drugs and treatments, such as cisplatin, irinotecan and radiation, which are still far to be a definitive cure by themselves. In this light, p38α inhibitors could represent suitable candidates. Indeed, they show a potential therapeutic effect against inflammation—and hence a potential preventive action against CRC formation, at least in the case of IBD-derived CRCs—and can boost an autophagic response at early stages of CRC development (Figure 1). Moreover, in combination with chemotherapy they can elicit autophagy induction, ultimately leading to cell death and tumor growth suppression (Figure 1).
